# On the Use of the Trephine in Injuries of the Head

**Published:** 1849-12

**Authors:** Thomas Lipscomb

**Affiliations:** Shelbyville, Tenn.


### WESTERN JOURNAL

OF

### MEDICINE AND SURGERY.

DECEMBER, 1849.

Art. I.—*On the Use of the Trephine in Injuries of the Head.* By Dr. Thomas Lipscomb, of Shelbyville, Tenn.

I propose, in the present paper, submitting a few reflections on the treatment of injuries of the head, and especially in reference to the use of the trephine, the time at which it should be resorted to, and the peculiar circumstances demanding its use. I may find it necessary briefly to refer to a few cases for the purpose of more clearly illustrating my views, and justifying the practice recommended.

At a very early period of my professional career I was induced by observation and reflection to call in question the soundness of the current doctrine in reference to the use of the trephine, the period at which it was proper, and when it was not allowable to use it, as also the particular circumstances demanding its application. I could not believe that a class of injuries possessing so much interest had been too lightly passed over. I was already aware that the views of medical or surgical writers did not always harmonize, but, on the contrary, opinions are held and doctrines taught on other subjects directly opposite, and that by authors of equal respectability.

But notwithstanding this general diversity of opinion, respecting the use of the trephine there was an extraordinary unanimity among authors. In this country, the unrivalled Physic had laid down principles of practice in which all his brethren appeared to concur. I believe the same was the case among the most distinguished surgeons in Europe. Samuel Cooper, Abernethy, Sir Charles Bell and Sir Astley Cooper were all agreed. Highly as I appreciate the character of these great lights of surgery, and much as I may admire their splendid achievements, yet I apprehend we may err in receiving too implicitly what they teach, and that we may possibly venerate them so much as to prevent inquiry into the correctness of their opinions or the soundness of their practice. This would be a fatal mistake, and yet it is one too often committed. We should not forget that the march of science is progressive, and that truth can only be attained by searching investigations. No authority is too high to be subjected to the most scrutinizing examination. Having a just appreciation of the value of human life and a proper sense of the responsibilities that attend us at every step, we cannot rest satisfied with the ipse dixit of any man. The opinions of our distinguished predecessors are entitled to great weight, and should ever be most respectfully deferred to; but not received as conclusive. On the contrary, we should weigh their opinions, carefully compare them with facts, reason, reflect, and then determine for ourselves.

What is the doctrine above referred to, which, until very recently, has been taught in Europe and in the United States for the last half century, or longer, in reference to the use of the trephine? I understand it to be this:—That we are not authorized to trepan, no matter what the character or extent of the cranial fracture, unless coma, stertorous breathing, &c., in short, unless those symptoms that result from compression of the brain, exist.—Indeed, should these symptoms be present we must endeavor to relieve them by drawing blood from the arm, emptying the bowels, applying cold to the head, keeping it elevated, &c. Should these means fail to mitigate the symptoms, we may resort to the trephine. For the correctness of this representation I refer to Dorsey's Surgery, Gibson's Surgery, Cooper's First Lines, Abernethy's works, &c.

I am fully aware that, previously to the period alluded to, there was too great a fondness for the use of the trephine; that, as has been observed by another, there was a time when he who could boast of having made the most perforations through the cranium was considered the best and most dexterous surgeon. Happily for the interests of science and the well-being of this class of patients, this season of error has passed away. But in avoiding one extreme we have run into the other; in shunning the errors of a former age, we have fallen into a practice by no means free from serious objections.

We should never lose sight of the force of the old axiom, that precaution is better than cure; then why wait, when meeting with an extensive fracture, with the bone shattered and depressed, and always more extensively fractured internally than externally?—I say, why should we wait for the occurrence of inflammation in the brain and its membranes, before attempting a removal of the cause from which it so frequently results: namely, the rough edges and sharp spicula of the fractured and depressed bone, against which the meninges of the brain are continually playing? Professor Gibson remarks, that should inflammation terminate in suppuration, in spite of bloodletting, leeching, purging, and even a large blister to the scalp, and give rise to symptoms of compressed brain, the trephine should be resorted to; but that the patient's chance of recovery is then very limited. Knowing, then, that it is infinitely easier to prevent inflammation than to subdue it, and that every spiculum of bone against which the membranes of the brain are forced produces a point to which an increased flow of fluids will be induced, the true practice would seem to be to remove the sources of irritation; for though at first it may be only a point or two, how soon the whole of the dura mater and other membranes, and even the brain itself, may be involved, none can tell. It is well known that inflammation passes readily to contiguous parts, and that once set up in the brain or its membranes, it is of a most formidably character.

It is admitted that, even in extensive fractures with considerable depression, we may by an active antiphlogistic course, succeed, for a time, in keeping down inflammation; but sooner or later this may be expected to ensue. A few cases will illustrate my views on this subject.

*Case* 1. T., aged about nine years, was thrown from a horse with his head against the end of a fence-rail, fracturing the superior portion of the right parietal bone extensively, but producing only a slight wound of the scalp. My friend, Dr. Barksdale, was called to see him, and finding only slight coma existing, he drew blood from the arm, gave him a saline cathartic, directed his head to be kept elevated and constantly cool, and the saline purgatives to be repeated *pro re nata*, and antimonials to be used as the degree of excitement might require—in short, treating the case efficiently and judiciously, according to the highest authorities. By the diligent use of these means, febrile excitement was kept down for a week; but in a day or two more, pains in the head ensued; then partial paralysis, and finally a convulsion. At this period, seeing that nothing less than an operation would avail, Dr. B. invited me to accompany him, and assist him in trepanning. Upon dividing the scalp, the fracture was found far more extensive than had been supposed, and the membranes of the brain beneath were highly inflamed. Upon elevating the depressed bone there was very considerable hemorrhage for a short time, and, in consequence of the exhausted condition of the patient from the effect of the active antiphlogistic course that had been pursued, he succumbed from the loss of an amount of blood which might have been harmless previous to his being subjected to these therapeutic measures, and died in a few hours.

*Case* 2. In July, 1834, when I had enjoyed but little experience in the treatment of cases of surgery, I was called, in company with Dr. P. Frazer, to see a man who, in an affray, when intoxicated, had been stricken on the left side of the head with a rifle, by which blow he was knocked down and partially stunned. The blow produced a considerable flesh wound, and an extensive fracture of the anterior portion of the left parietal bone, with considerable depression. On our arrival we found he had recovered from the concussion, and was a little comatose, or heavy; but he answered questions intelligibly, and, notwithstanding the appearance of the wound seemed to require an operation, the constitutional symptoms did not appear to justify it; so, after advising a cathartic, low diet, and keeping his head cool, and insisting on being informed of any considerable change, we left him. Our course, however, was not satisfactory to his friends, who sent for another physician, and, at his request, we were again called in. On our arrival, we found him about as we had left him, still rational and capable of conversing intelligibly. Our medical friend decided that an option was necessary, but as the weight of authority was opposed to the trephine under the circumstances, after consulting together for a time, we agreed, by way of compromise, to divide the scalp down to the bone, and then make a second incision across the first, and dissect up the scalp so as to expose the fracture fully. If it should appear that trepanning would certainly, in the end, become necessary, then we would go on and complete the operation. This course met with the approbation of the friends. Upon exposing the bone, the fracture was found to be a very extensive one, comminuted in character, and the inner table depressed to such an extent that it appeared inexplicable how the intellectual functions were in any degree preserved. It then appeared, and so I now believe, that every hour's delay was an injudicious postponement of what was inevitable in the end. Entertaining this view, the trephine was used, the bone elevated, and our patient recovered without an unpleasant symptom. This case made a powerful impression on my mind.

*Case* 3. About a year and a half after the occurrence of the last case, I was called to I. F. S., a youth of eleven or twelve years of age, who had just received a kick from a horse, newly shod. The superior portion of the left parietal bone received the blow, and from the very small wound in the scalp, I suppose that only one heel of the shoe touched the head. The boy was knocked down and suffered momentary insensibility, from which he had entirely recovered before I saw him. There was no stupor, nor coma, nor mental derangement, and, as just observed, a very small wound in the scalp, which did not extend into the cranium. But there was a considerable depression of bone, and as I supposed the fracture might ultimately require an operation, and the distance was short, I requested a consultation with Dr. Barksdale, who, after examining the case, coincided with me in opinion.—We proposed to Mr. S., the father of the boy, to divide the scalp through the wound, and, by another incision across the first one, prepare to detach the scalp sufficiently to see the extent and peculiar character of the fracture. He reluctantly consented to this course, which was immediately adopted, when we found a fine specimen of stellated fracture, and the centre of the fracture depressed so much that it was evident there must be a rough point against which the brain and membranes would be continually forced. In addition to this, the bone was shattered so much that there was not a remote probability that a reunion of the broken portions could take place; and scarcely a doubt that the trephine would be required in a few days. Thus, feeling assured that there was almost a certainty as to the inevitable necessity of the trephine, and that even if, contrary to our settled convictions, it should not be required, months of anxiety would be endured until the tedious process of exfoliation was completed, we did not hesitate to trepan, and remove the shattered bone, as well as elevate the depressed portion which was not so completely broken up. He bore the operation well, and had a most happy recovery.

I might advert to a number of other cases bearing on the point in question, but perhaps the time might not be profitably spent, and so I forbear.

From the foregoing facts, I infer that if the subject of the first case had been operated upon at an earlier period, and before the vital energies were so completely exhausted, his chance of recovery would have been greatly enhanced. I infer, also, that, although the case was treated in accordance with the standard authorities on the subject, it would have been a better practice to have divided the scalp by the crucial or tripod incision, at the first visit, and thereby have fully exposed the fracture, when it would have been seen that the trephine was absolutely necessary.

In reference to Cases 2 and 3, I infer, that had no enlargement of the wound been made, and no detaching of the scalp, but an effort by a rigid antiphlogistic course to keep down inflammation in the brain and membranes, the success would only have been partial, and meningitis, as well as cerebral inflammation, would have been set up, and an ultimate resort to the trephine rendered necessary; and that at a period and under circumstances which would have made recovery very doubtful.

In the third place, I infer, that the evils of trepanning have been overrated by our standard authors.

I also infer that, notwithstanding we may frequently ward off, for a time, coma, convulsions, &c., as consequences of depressed bone, by the use of active depletory means, we leave the patient in a condition more liable to those affections than he would be either to them or any other evil effects from the use of the trephine.

I infer that this is pre-eminently the case with young subjects, with whom, if the bone is considerably depressed, and left in that condition, the natural growth of the head increases that depression, and the evils likely to arise from it, to wit: epilepsy, convulsions, &c. In support of this inference I need only direct attention to the great number of cases that have occurred to, and been relieved by, surgeons of extensive practice. Prof. Gibson was in the habit, a few years ago, of detailing a case of this kind to his class. A man became subject to epilepsy from an injury received in childhood, and of which disease he was cured by trepanning eighteen years after the receipt of the injury.

And the general inference which I deduce from the whole is, that we should, in all cases of fracture with evident depression of bone, incise and detach the scalp sufficiently to examine the fracture as to its extent and peculiarities; and if we find it such an one, as is almost certain to require an operation ultimately, then proceed at once and complete it, whether coma or stertorous breathing exist or not. To this position my observations and reflections have led me; and this course I confidently recommend to the profession as the one most compatible with the safety of this class of patients. I do so with due deference to those illustrious authorities in Europe and America by whom a different course is recommended.

After determining to present my views to the profession on this subject, I found, upon examining the Elements of Surgery, by Robert Liston, fourth American edition, with notes by Professor Gross, (page 234,) that a resort to the trephine is recommended by that distinguished surgeon for certain descriptions of fracture before inflammatory symptoms have set in; and that under certain circumstances he considers it right to divide the integuments for the purpose of fully exposing the fracture.—And I infer from the absence of any remarks by the American editor that he entertains similar views. This being the case, I think the interests of humanity would have been promoted by a recommendation, more direct, under the sanction of his influential name.

Shelbyville, Tenn., Oct. 20, 1849.

